# Supplementary material for: Autocrine TGFβ1 Opposes Exogenous TGFβ1-Induced Cell Migration and Growth Arrest through Sustainment of a Feed-Forward Loop Involving MEK-ERK Signaling
Source: Cancers (Basel). 2021 Mar 17;13(6):1357. doi: 10.3390/cancers13061357 (PMC8002526; doi:10.3390/cancers13061357)
Supplement: Supplementary file 1 [file cancers-13-01357-s001.zip › supp proof/cancers-1104178 re-revised supp.docx]

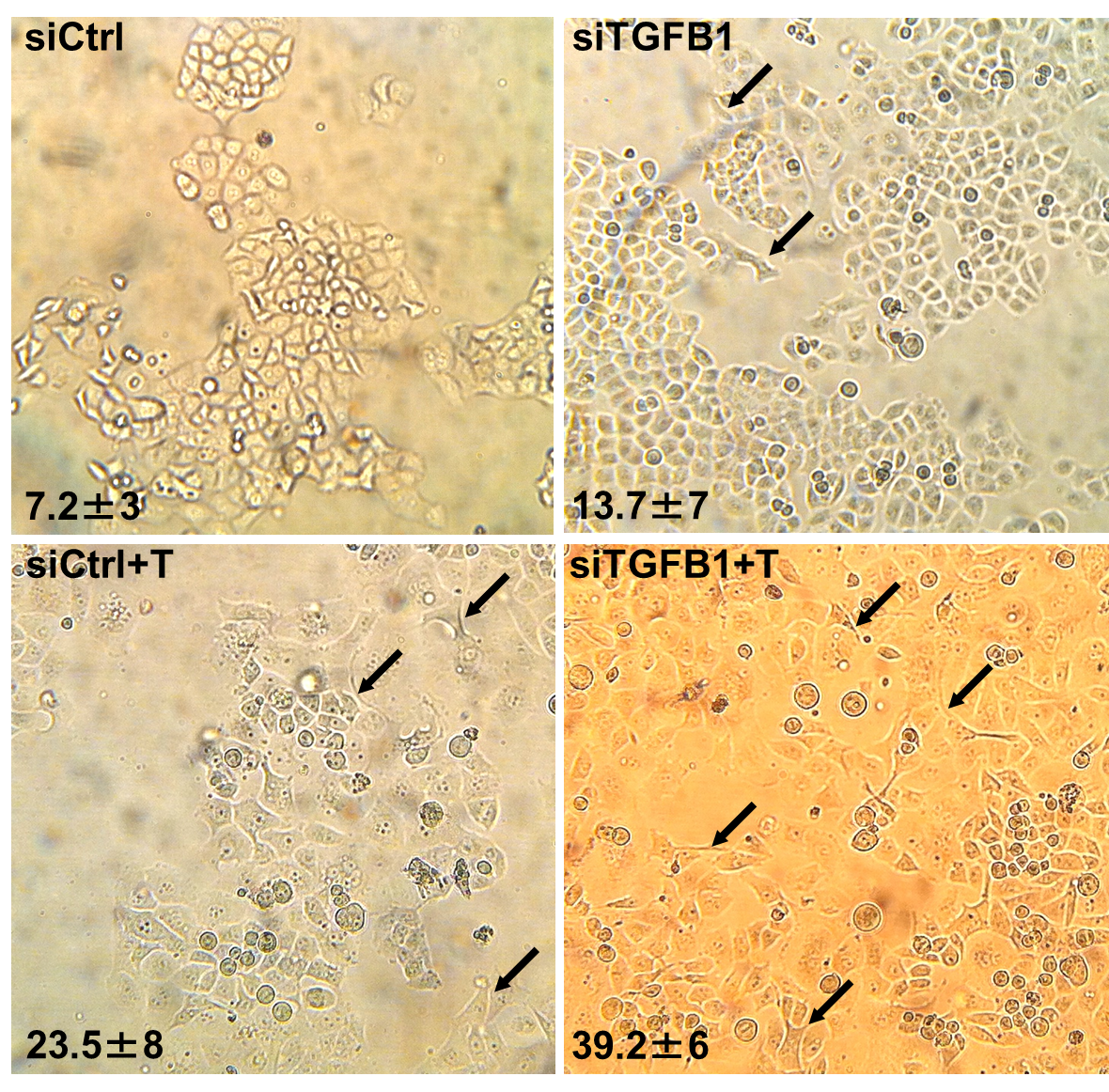


**Figure S1.** Effect of knockdown of *TGFB1* on rhTGFβ1-induced changes in cell morphology of Panc1 cells. Phase-contrast images of Panc1 cells transfected with an irrelevant control siRNA (siCtrl) or siRNA to TGFB1 (siTGFB1) and subsequently left untreated or treated for 24 h with 5 ng/ml rhTGFβ1 (T) in medium with 1% FBS. Three experiments were performed in total, of which a representative one is shown. The arrows point to cells with an elongated morphology. Insets indicate the percentage of spindle-shaped cells per visual field (mean ± SD, n=3, independently counted by two investigators in a blinded fashion), *p* = 0.0073 for siCtrl+T *vs*. siTGFB1+T and *p* = 0.009 for siTGFB1 *vs*. siTGFB1+T. Magnification: x200, *bar:* 25 µm.

Rel. TGFβ1 conc.

Ctrl

0

20

40

60

80

100

TGFB1

*

*

*

Panc1

MDA-MB-231

Ctrl

0

20

40

60

80

100

*

*

**Figure S2.** Validation of successful TGFB1 knockdown in Panc1 and MDA-MB-231 cells. Panc1 or MDA-MB-231 cells transfected with either control (Ctrl) siRNA or *TGFB1* siRNA were allowed to condition their serum-reduced (1% fetal bovine serum) growth media for 24 h. Samples of conditioned media were subjected to ELISA measurement of total (bioactive + latent) TGFβ1. Data are the mean ± SD of triplicate samples from the cells analyzed in Figure 1. *** *p* < 0.001, ** *p* < 0.01.

TGFβ1 content

(ng/µg protein/24h

0

1

2

3

4

5

*

Panc1

V

pTGFB1

*

TGFβ1 content

(ng/µg protein/24h

0

1

2

3

4

5

MDA-MB-231

V

pTGFB1

*

*

- rhTGFβ1

+ rhTGFβ1

- rhTGFβ1

+ rhTGFβ1

**Figure S3.** Content of TGFβ1 in culture supernatants from Panc1 and MDA-MB-231 cells with ectopic TGFβ1 expression and added rhTGFβ1. Panc1 or MDA-MB-231 cells transfected with either empty vector (V) or an expression vector for TGFβ1 (pTGFB1) were allowed to condition their serum-reduced (0.5% FBS) growth media for 24 h. Aliquots of conditioned media with or without the addition of rhTGFβ1 (5 ng/ml) were subjected to ELISA measurement of total (bioactive + latent) TGFβ1. Data are representative of three experiments (mean ± SD of triplicate samples from the cells analyzed in Figure 3). * *p* < 0.05.

26

17

M

p21^WAF1^


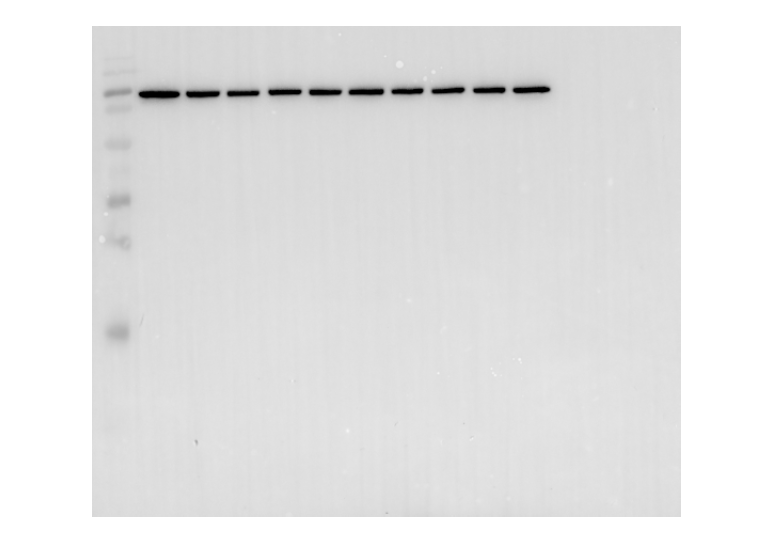


HSP90

95

0

8

24

rhTGFβ1 (h)


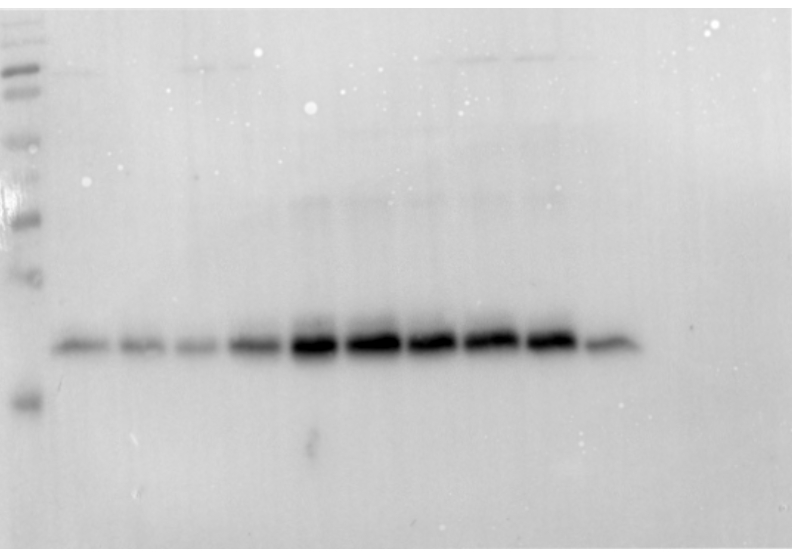


**Figure S4.** Induction of p21^WAF1^ by rhTGFβ1 in breast epithelial cells. MDA-MB-231 cells were left untreated or were treated for the indicated times with rhTGFβ1 (5 ng/ml). After treatment, cell lysates (in triplicate) were subjected to immunoblotting for p21^WAF1^, and HSP90 as a loading control. The numbers on the left denote the size of the bands of the molecular weight marker (M).


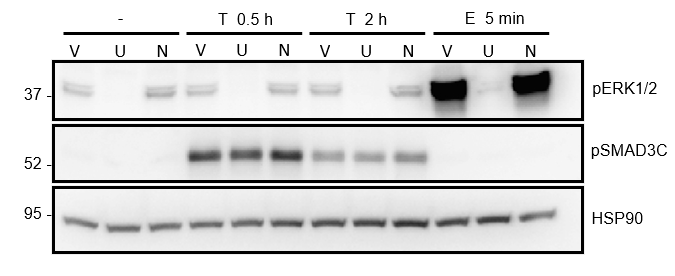


**Figure S5.** U0126 blocks activation of ERK1/2 but not rhTGFβ1-induced activation of SMAD3. Panc1 cells were grown to confluence, starved for 24 h in medium containing 0.1% bovine serum albumin and left untreated or were treated for the indicated times with rhTGFβ1 (T, 5 ng/ml) in the absence or presence of either vehicle (V, 0.1% DMSO), the MEK inhibitor U0126 (U, 20 µM), or the Rac1 inhibitor NSC23766 (N, 200 µM). The latter drug (used as a negative control) failed to prevent ERK1/2 phosphorylation although RAC1 can act as an upstream activator of ERK1/2 in the growth of transformed cells [58]. Cells were subjected to immunoblotting for pERK1/2, pSMAD3C, and HSP90 to control for equal loading. The functionality of U0126 was confirmed by its ability to block ERK1/2 activation after a 5 min challenge of cells with EGF (E, 10 ng/ml).


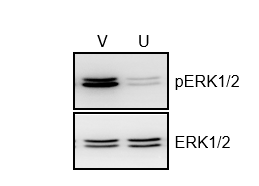


**Figure S6.** The MEK inhibitor U0126 inhibits ERK activation in MDA-MB-231 cells. MDA-MB-231 cells were treated with either vehicle (V) or 10 µM U0126 (U) for 24 h in normal growth medium followed by lysis and sequential immunoblotting for phospho-ERK1/2 (pERK1/2) and total ERK1/2.


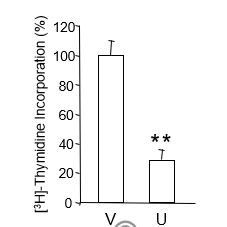


**Figure S7.** Inhibition of ERK activation inhibits proliferation in MDA-MB-231 cells. MDA-MB-231 cells were treated with either vehicle (V) or 10 µM U0126 (U) for 24 h in normal growth medium. During the last 4 h of the stimulation period cells were pulsed with [^3^H]-thymidine and further processed as described in the Methods section.


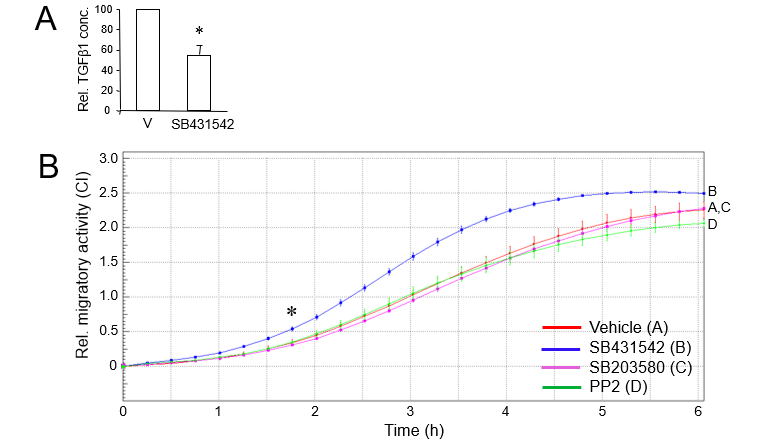


**Figure S8.** Effects of SB431542 on endogenous TGFβ1 secretion and cell migration in TNBC cells. (**A**) MDA-MB-231 cells were pretreated with SB431542 (1 µM) or vehicle (V) for 1 h in normal growth medium. This medium was then removed and replaced by medium containing 0.5% FCS and either 1 µM SB431542 or vehicle. Cells were allowed to condition the medium for 24 h. Culture supernatants were harvested and subjected to ELISA of TGFβ1. Data (mean ± SD from triplicate wells) were normalized to the protein content in the same well and given relative to the concentration (conc.) of TGFβ1 in the V control set at 100%. Data shown are representative of three assays. (**B**) MDA-MB-231 cells were subjected to real-time cell migration assay in the presence or absence of SB431542 (1 µM), SB203580 (1 µM), or PP2 (10 µM). A representative assay from s a series of three independent assays is shown (mean ± SD of triplicate wells). Differences between the red curve (tracing A) and the blue curve (tracing B) were significant at 1:45 (*) and remained so until termination of the assay.

**Table 1.** Primers used for qPCR.

| **Designation** | **Sequence (5’→3’)** |
| --- | --- |
| F2RL1-sense | ACTCCAGGAAGAAGGCAAACA |
| F2RL1-antisense | TGGTCTGCTTCACGACATACA |
| GAPDH-sense | TTGCCATCAATGACCCCTTCA |
| GAPDH-antisense | CGCCCCACTTGATTTTGGA |
| SERPINE1-sense | CTTCTTCAGGCTGTTCCGGAGC |
| SERPINE1-antisense | GGGTCAGGGTTCCATCACTTGG |
| SNAI2-sense | ATATTCGGACCCACACATTACCT |
| SNAI2-antisense | GCAAATGCTCTGTTGCAGTGA |
| SNAI1-sense | CTGCTCCACAAGCACCAAGAGTC |
| SNAI1-antisense | CCAGCTGCCCTCCCTCCAC |
| TBP-sense | GCTGGCCCATAGTGATCTTT |
| TBP-antisense | CTTCACACGCCAAGAAACAG |
| TGFB1-sense | cccAGCATCTGCAAAGCTC |
| TGFB1-antisense | GTCAATGTACAGCTGCCGCA |
