# Supplementary material for: Autocrine TGFβ1 Opposes Exogenous TGFβ1-Induced Cell Migration and Growth Arrest through Sustainment of a Feed-Forward Loop Involving MEK-ERK Signaling
Source: Cancers (Basel). 2021 Mar 17;13(6):1357. doi: 10.3390/cancers13061357 (PMC8002526; doi:10.3390/cancers13061357)

Uncropped blots: Figure 1C

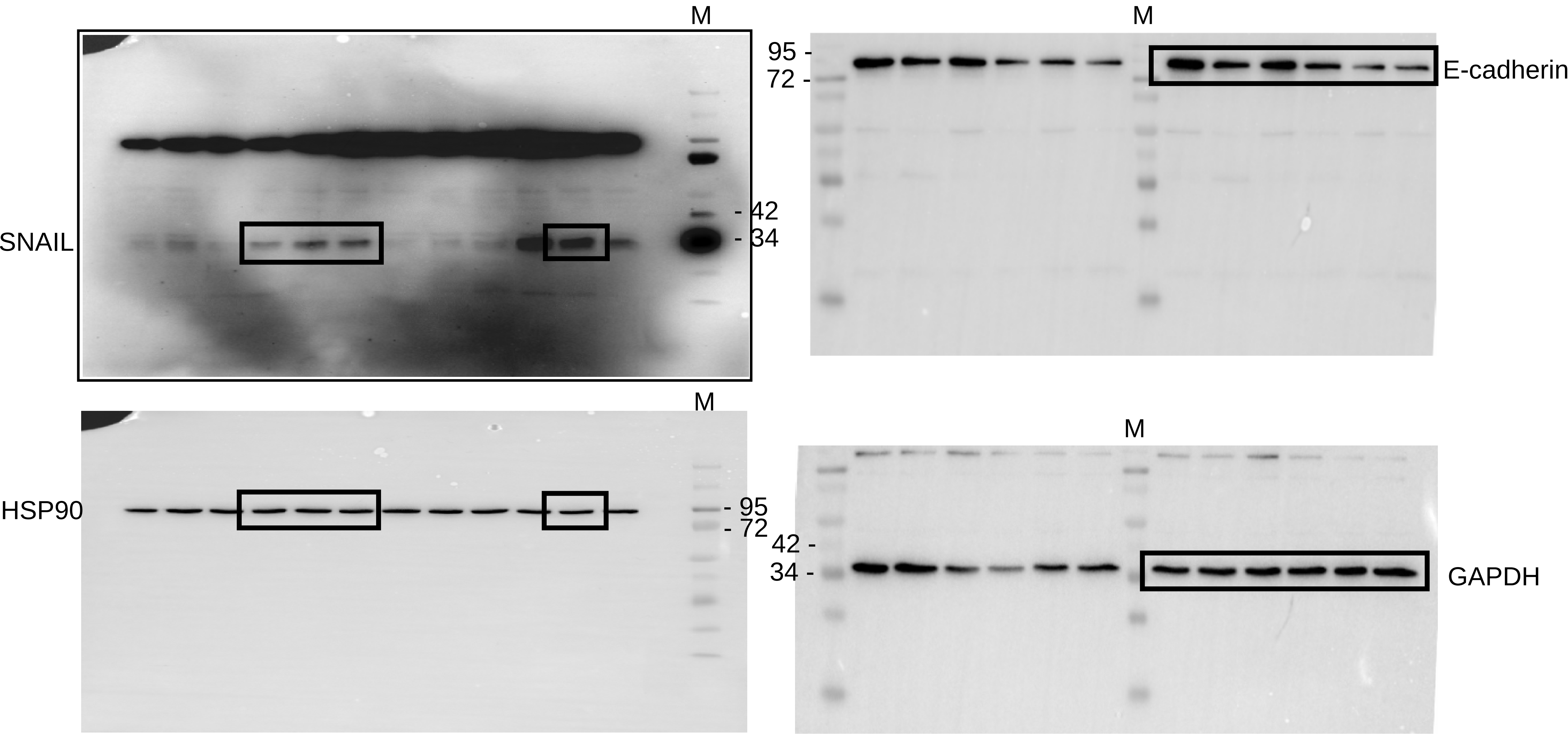

Figure legend: M = molecular weight marker (SM1841, Fermentas/Thermo Fisher Scientific

Uncropped blots: Figure 4B

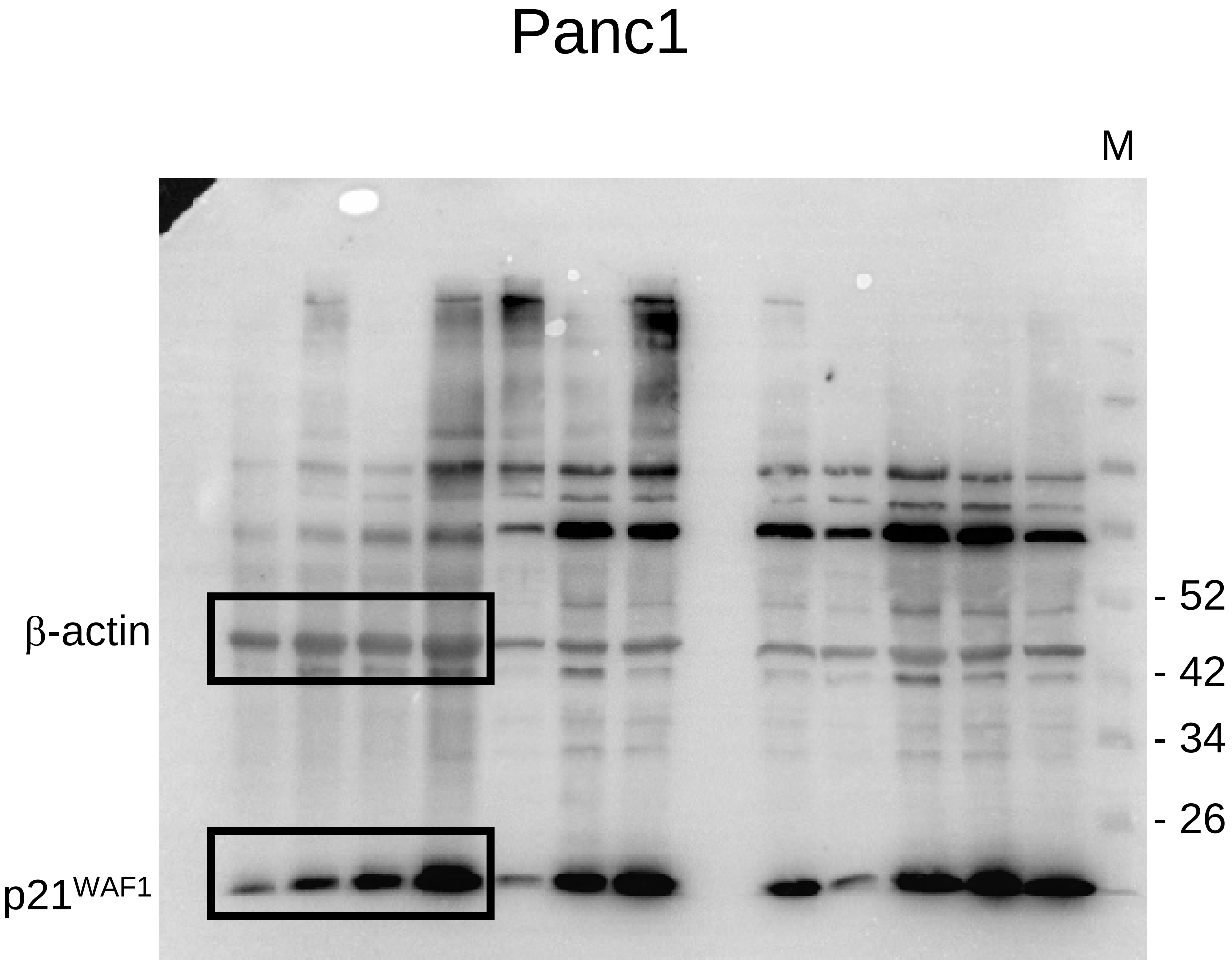

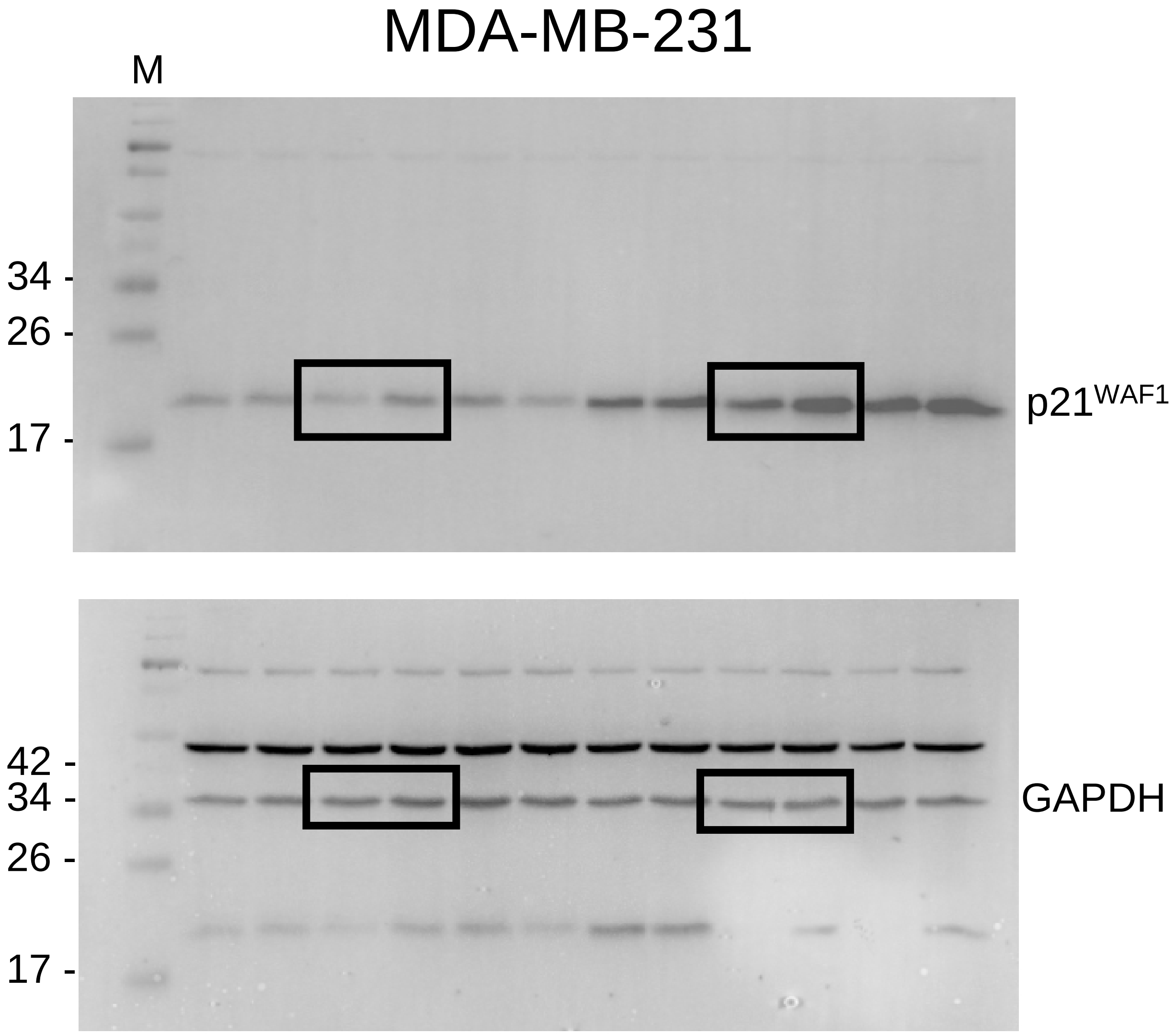

Uncropped blots: Figure 5D

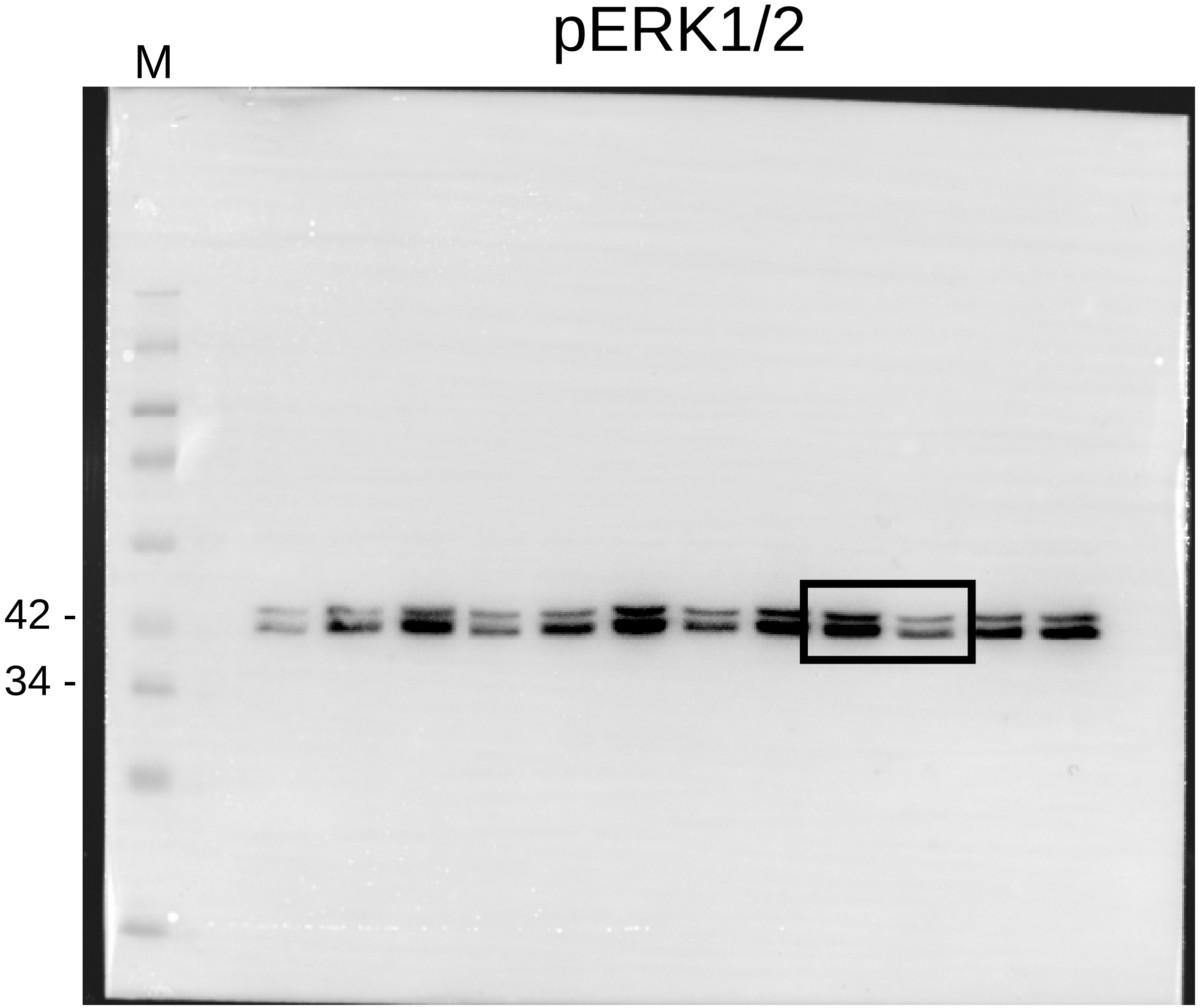

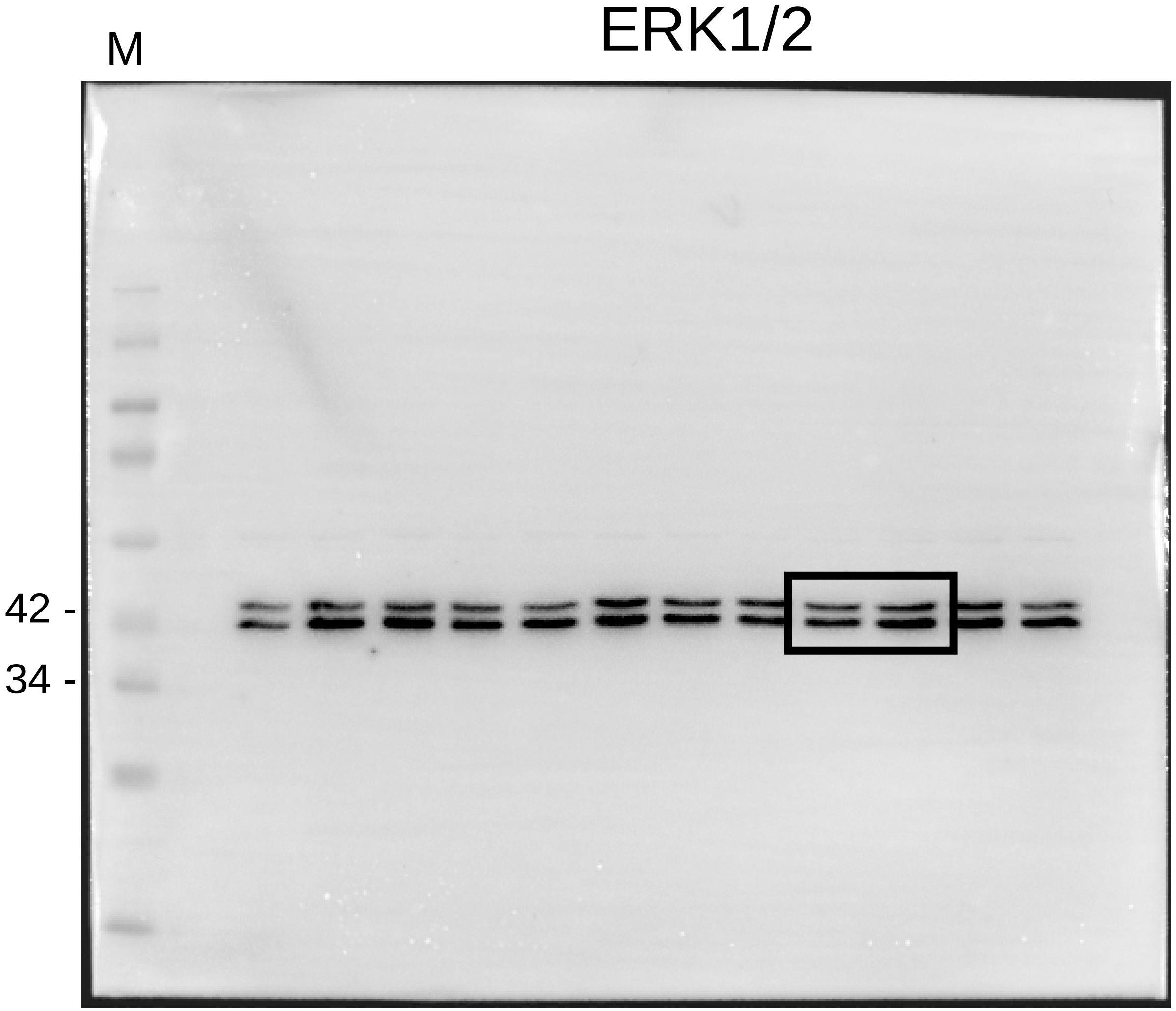

Uncropped blots: Figure 6B

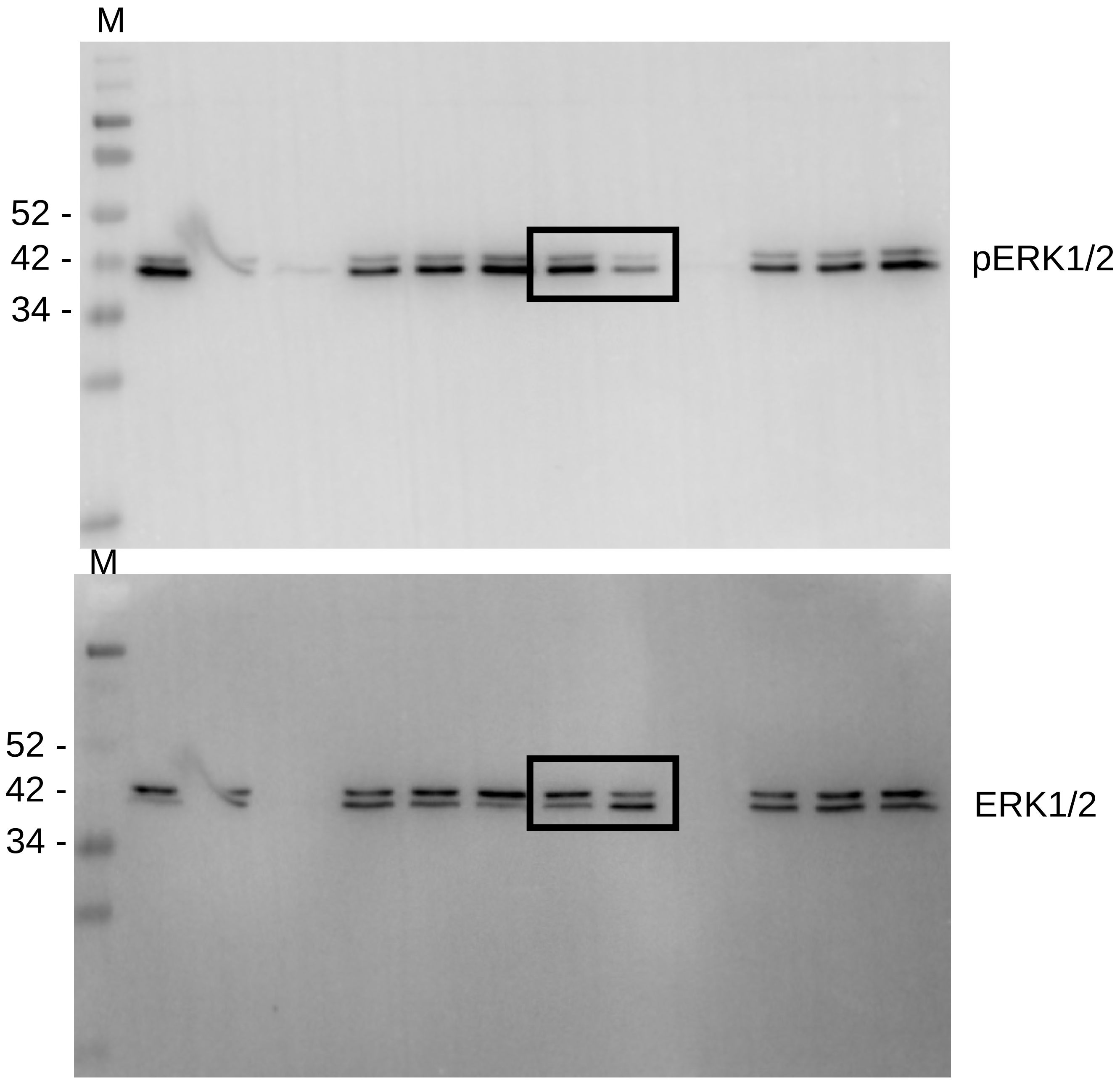

Uncropped blots: Figure S4

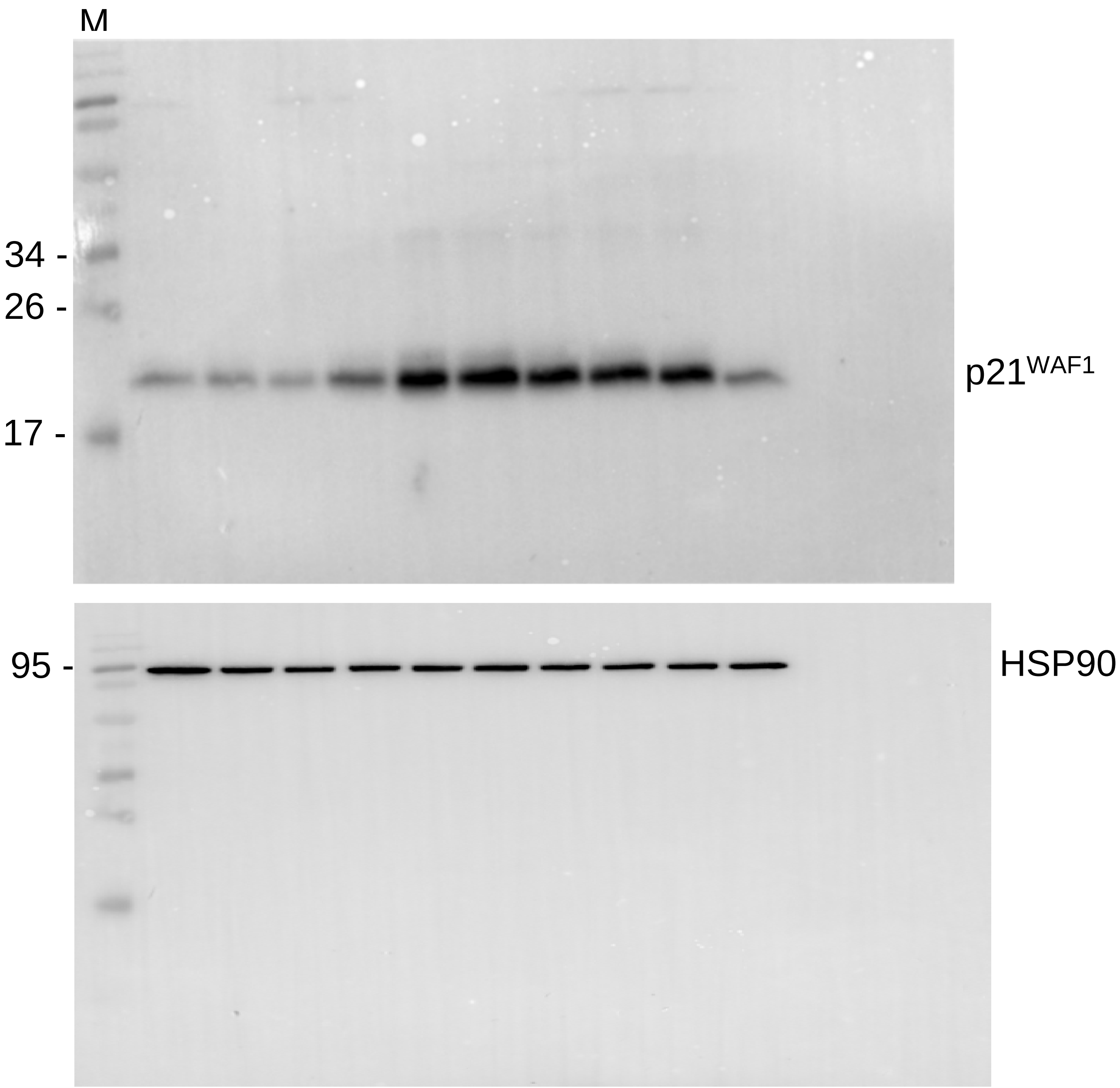

Uncropped blots: Figure S5

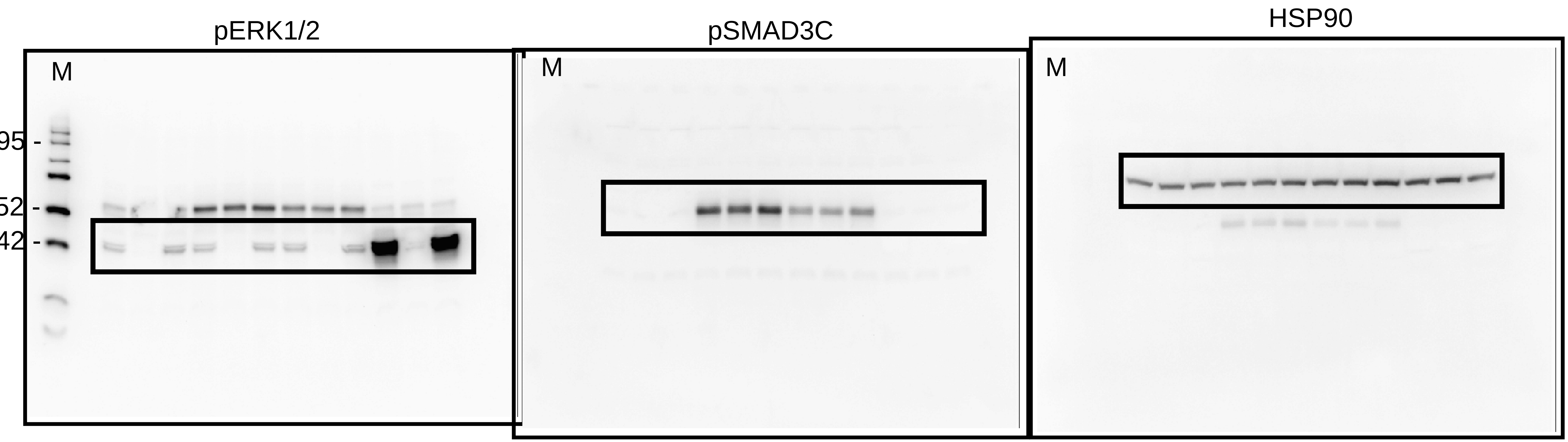

Uncropped blots: Figure S6

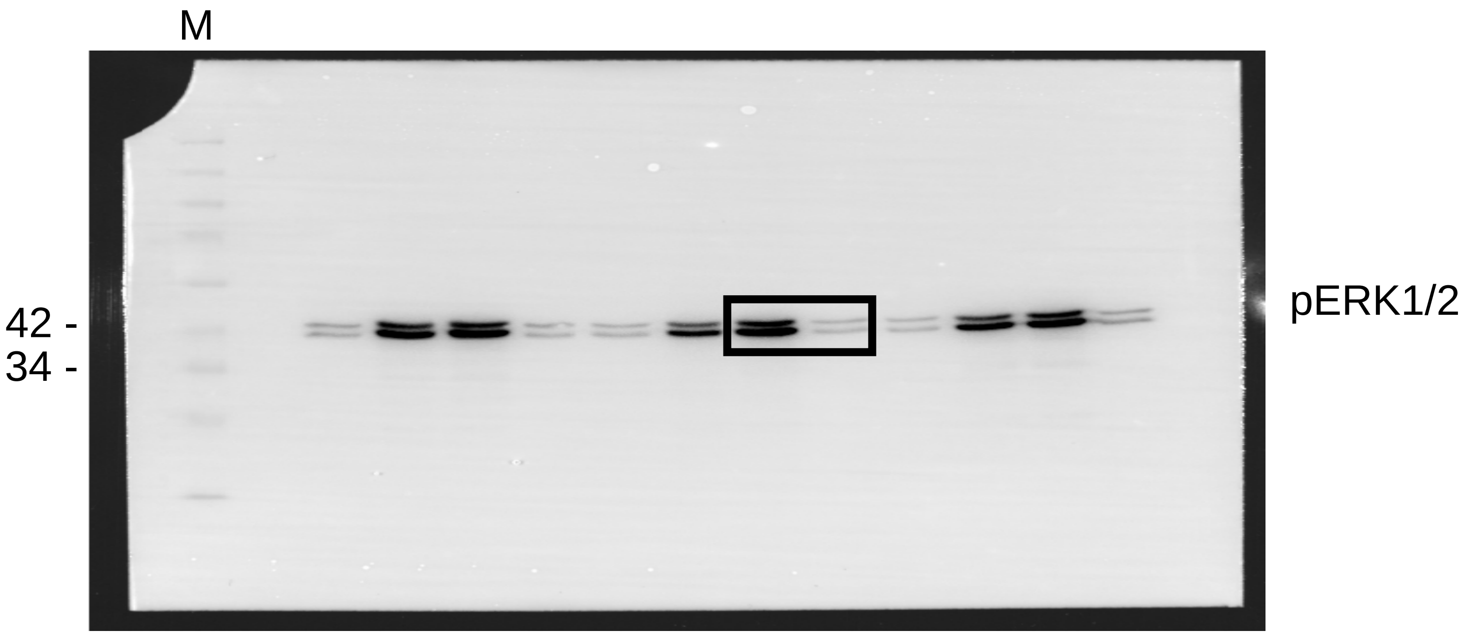

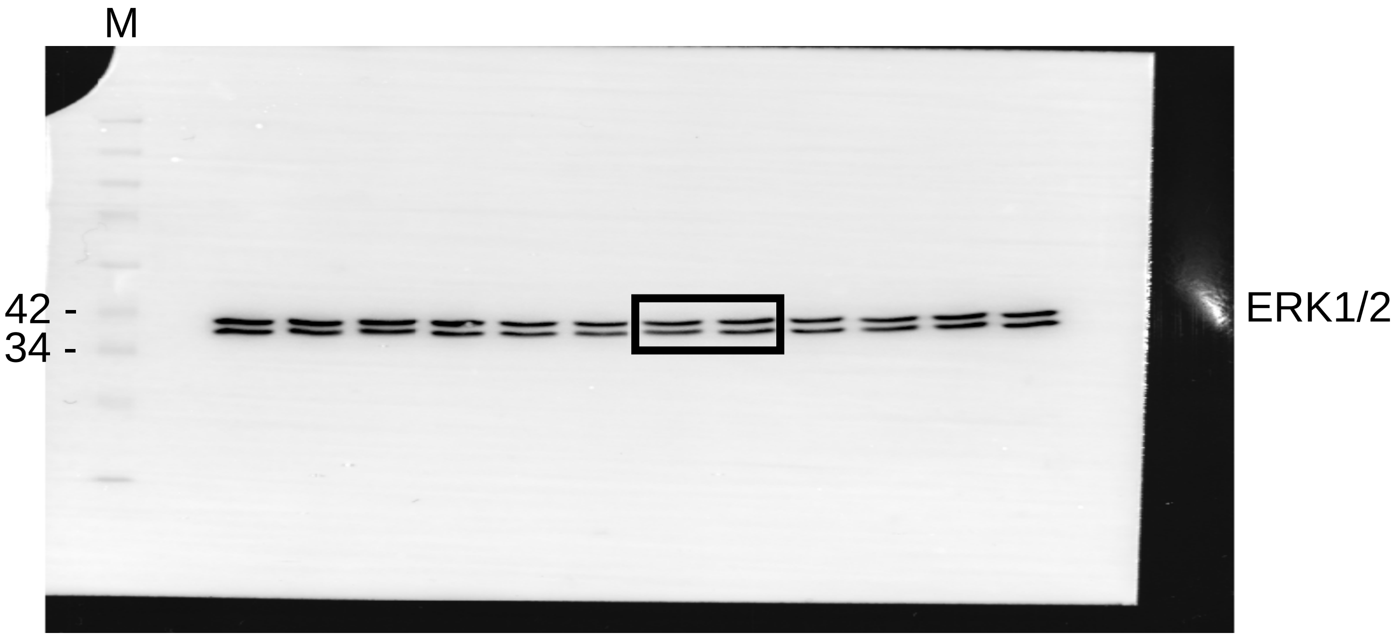

Supplement: Supplementary file 1 [file cancers-13-01357-s001.zip › supp proof/Uncropped blots_revised.pdf]
